# Why Business Modeling is Crucial in the Development of eHealth Technologies

**DOI:** 10.2196/jmir.1674

**Published:** 2011-12-28

**Authors:** Maarten van Limburg, Julia EWC van Gemert-Pijnen, Nicol Nijland, Hans C Ossebaard, Ron MG Hendrix, Erwin R Seydel

**Affiliations:** ^1^Center for eHealth Research and Disease ManagementDepartment of Psychology, Health and Technology, Faculty of Behavioural SciencesUniversity of TwenteEnschedeNetherlands; ^2^National Institute for Public Health and the Environment (RIVM)BilthovenNetherlands

**Keywords:** Business model, cocreation, collaboration, eHealth, implementation, multidisciplinary, stakeholder, sustainability, value creation

## Abstract

The impact and uptake of information and communication technologies that support health care are rather low. Current frameworks for eHealth development suffer from a lack of fitting infrastructures, inability to find funding, complications with scalability, and uncertainties regarding effectiveness and sustainability. These issues can be addressed by defining a better implementation strategy early in the development of eHealth technologies. A business model, and thus business modeling, help to determine such an implementation strategy by involving all important stakeholders in a value-driven dialogue on what the technology should accomplish. This idea also seems promising to eHealth, as it can contribute to the whole development of eHealth technology. We therefore suggest that business modeling can be used as an effective approach to supporting holistic development of eHealth technologies. The contribution of business modeling is elaborated in this paper through a literature review that covers the latest business model research, concepts from the latest eHealth and persuasive technology research, evaluation and insights from our prior eHealth research, as well as the review conducted in the first paper of this series. Business modeling focuses on generating a collaborative effort of value cocreation in which all stakeholders reflect on the value needs of the others. The resulting business model acts as the basis for implementation. The development of eHealth technology should focus more on the context by emphasizing what this technology should contribute in practice to the needs of all involved stakeholders. Incorporating the idea of business modeling helps to cocreate and formulate a set of critical success factors that will influence the sustainability and effectiveness of eHealth technology.

## Introduction

Health care systems worldwide will face sustainability problems in the near future caused by a tension between an increasing demand for and a mismatch in the supply of health care services [1]. The growing demand for health care services is generally explained by an aging population and the rise in prevalence and incidence of chronic diseases and obesity. In addition, these increased demands imply increased complexity of treatments due to rapid advances in medical technology and increased comorbidity [1,2]. At the same time, the health care industry struggles with inefficiencies in procurement of supplies and inadequate use or lack of resources. In the United States, for example, the financial consequences of inefficiency are estimated to be in the range of 30% to 40% of total health care costs [3]. Without rapid action, health care services shall soon become less accessible and unaffordable and will deteriorate in quality.

In many industries, Web-based and mobile technologies have changed and are still changing conventional business activities to Internet-based activities such as Web 2.0 services or e-business [4,5]. In the health care industry, similar opportunities, often called eHealth, seem promising to help solve the aforementioned demand and supply problems in healthcare [6,7]. Indeed, eHealth technologies can contribute to improved communication and information sharing among health professionals, patients, and researchers and aim to improve quality and effectiveness of health care services [6,8,9]. However, eHealth technologies suffer from a range of recurring problems [3,10-16] as outlined in Textbox 1.

These problems can be attributed to insufficient attention to the development process and implementation of eHealth technologies. We believe that in order to tackle the aforementioned problems and to ensure a proper uptake, long-term sustainability, and effectiveness, new development frameworks are needed that make implementation an integral part of eHealth development. We see that implementation of eHealth technologies in practice is underestimated and overlooked in eHealth development approaches. Therefore, we proposed a new holistic approach in our paper, “A Holistic Framework to Improve the Uptake and Impact of eHealth Technologies” [17], which describes the entire development and is aimed at creating a fit between technology, humans, and organizations.

**Recurring Problems of Ehealth Technologies**

· Currently established financial structures slow down innovation.

· Necessary legislations for modernizing health care lag behind.

· Involved parties are reluctant and uptake remains low.

· eHealth development focuses too strongly on engineering-driven solutions.

· eHealth technologies are deployed in a fragmented fashion and have poor scalability.

· The number of stakeholders and dependencies cause complexity.

· There is a lack of cost-effectiveness studies.

· eHealth research tends to focus on finding clinical evidence in terms of health outcomes, for example, yet the impact of eHealth technology does not rely solely on clinical evidence; there are more factors that determine the success of eHealth technology.

## CeHRes Roadmap

**Figure 1:**
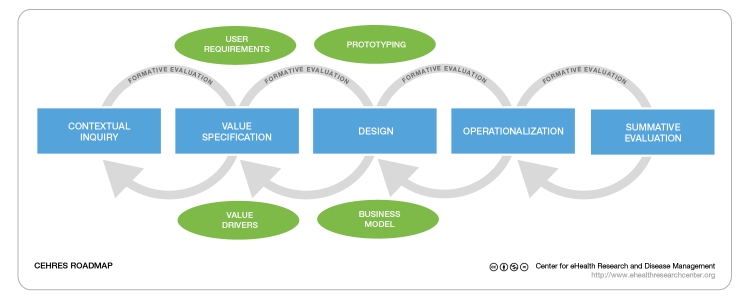
CeHRes Roadmap.

The Center for eHealth Research and Disease Management (CeHRes) Roadmap (Figure 1), introduced in “A Holistic Framework to Improve the Uptake and Impact of eHealth Technologies” in this issue of the *Journal of Medical Internet Research* [17], offers a holistic approach to eHealth development. This roadmap guides the development of persuasive technology and business modeling as interwoven activities. This approach allows eHealth technologies to be designed according to the needs of its users and to fit with their behavior, but also, due to business modeling, it allows the development process to be value-driven. Stakeholders are involved in the development process and, based on their values, an eHealth technology can be designed matching with intended collaboration and cocreation, and eventually an implementation can be found.

## Why eHealth Needs Business Modeling

In this paper we focus on business modeling and why it supports the development of eHealth technologies. Business modeling is interwoven with development to make both design and implementation value-driven. After all, it is futile to develop an eHealth technology that does not catch on because in practice it does not match demands or its intended purpose.

Implementation must ensure that an eHealth technology will live up to its fullest potential in real-world conditions and circumstances. In order for eHealth technology to succeed, all organizations have to collaborate and interact, and some organizations have to maintain and perhaps fund the project. eHealth technology needs to fit in existing care infrastructures or, perhaps even more importantly, be a catalyst for new, innovative care infrastructures. In other words, eHealth development encompasses more than technical design. It requires additional research to determine an implementation strategy, that is, a plan to embed technology in its intended practice. Implementation starts with detecting and involving concerned parties and results in a business model that describes the value creation and acts as the basis for a care infrastructure for collaboration and cocreation, possibly with multiple organizations involved. To our knowledge, very few implementation rationales relating to eHealth technologies have been explained. Many of these eHealth technologies are developed with a “jump on the eHealth bandwagon” mentality without clear predetermined goals. Once an eHealth technology has been developed and it becomes apparent that goals are needed, the organization finally starts to think about an implementation strategy. So, current eHealth implementations are usually done *post* development rather than integrated *in* the development process.

Attention to implementation appears too late in the development, and we therefore point out that it is crucial to start preparing an implementation strategy early on. It is better to invest more time and money in researching how eHealth technology can be implemented in its intended care practice than to invest money in an eHealth technology that will not have a satisfying uptake. It happens too often that as soon as research funding stops, an eHealth technology cannot be implemented sustainably, mainly because there is neither support nor interest from other parties. Through business modeling, development of eHealth technologies can be guided with a value-driven evaluation of what is necessary and what is not. Often eHealth technologies are built as replacements for or copies of existing care services and are then fine-tuned for user requirements using user- or human-centered design principles. It is yet to be questioned whether this approach is effective and whether the choices made are really grounded. Business modeling introduces research activities *before* the start of the actual technical design that focus on the context of eHealth technology and provide value drivers that will ground choices of what to develop.

## Starting With a Context

An important early step in the development of eHealth technology is analyzing the relevant problem, that is, an eHealth technology is meant to improve a problem of inefficiency or a lack of information or communication. In order to take proper action, the situation needs to be carefully assessed: this is known as *sensemaking* [18]. It is tempting, however, to rush toward thinking of technical solutions for a problem. Such fast solutions may lead to a solution that is technically state-of-the-art but poorly suited to the problem. By analyzing the problem at hand, eHealth technology will gain more context, and this increased understanding will contribute to all further choices that are required in the development process and the implementation. This is why the *contextual inquiry* in our business modeling approach is a crucial first step.

By discussing the problems with all concerned parties (so-called stakeholders, see next paragraph), it becomes clearer which parties will play an important role in the development process and which parties may come to play a role in the implementation of the eHealth technology. Also, this problem-oriented dialogue helps to make these parties more aware of each other’s problems, as health care organizations often have limited knowledge of the processes and/or problems that go on at other organizations. In fact, during several of our workshops, it became apparent that people even within the same organization were unaware of each other’s exact responsibilities and duties (see Textbox 2 as example).

**Example Case: Finding the Problems With Antibiotics Prescription**

Our intention was to understand and improve the behavior behind antibiotics prescription as part of the contextual inquiry for an eHealth technology that is in development. Based on a literature review and expert interviews, we identified the general problems with imprudent antibiotics prescription (causing a high risk of infections), the general prescription process, as well as key stakeholders. We organized a workshop with these key stakeholders within the first hospital ward where we had aimed to start our pilot. These key stakeholders discussed the problems they face daily based on patient scenarios validated by infection experts. This workshop not only enlightened the project management (us) to what problems and opportunities there were, but also created awareness among stakeholders as to what problems *other* stakeholders face and how the mutual problems also affected others. This awareness is vital for the collaboration of these key stakeholders and their future commitment to the project.

## Stakeholder Participation

Everyone who affects or is affected by a project is considered a stakeholder [19]. It is therefore critical for the success of eHealth technology to understand the value needs of each stakeholder [20]. Through participation of stakeholders in the development process of eHealth technologies, value needs can be retrieved and a mutually determined fit can be found. According to Pagliari, developing eHealth technologies is a multidisciplinary process [21]. Business modeling deepens this multidisciplinary development of eHealth as it brings multiple stakeholders together in the discussion of the necessary implementation. Business modeling also allows for an exploration of the value needs of stakeholders that determines both the design of the technology as well as the implementation.

There are many types of stakeholders associated with eHealth: patients, policymakers, vendors, insurers, health care organizations and providers, home care workers, and employers [22]. Therefore, every eHealth technology will have its unique stakeholder network (sometimes also referred to as an ecosystem) that determines potential customer segments and the infrastructure required for value cocreation for eHealth technology. Patients are often overlooked as stakeholders, yet they also have to participate in eHealth development. Patients often use or are subjected to the technology and have legal and social rights to be part of the development [8]. Patient empowerment does not stop at letting patients use eHealth technology; patients should be invited to participate in the development process of technology as well.

The level of engagement determines the salience of each stakeholder to the stakeholder network [23]. In our roadmap, we start by mapping the stakeholder network as part of the contextual inquiry process. As suggested by Sharp, it is best to start with baseline stakeholders (in our approach we start with project initiators) and let them suggest more stakeholders that may be relevant to the eHealth project [24] (see Textbox 3 as an example). We base stakeholder salience on three variables: power, legitimacy, and the urgency of the stakeholder [25]. There are various ways to assess salience. This can be done either by asking experts to score the above variables or by asking the stakeholders to score each other. The next step is to start discussing value with stakeholders. The most salient stakeholders will eventually have a bigger influence on the value drivers than less salient ones.

**Example Case: Finding Stakeholders Through Experts and by “snowballing”**

In the early phases of any project, there are one or more initiators involved that can provide a list of baseline stakeholders. In one project, for example, a health information technology (IT) company wanted to develop a personal health record service. We spoke to several opinion leaders in health insurance, eHealth, and patient empowerment to form a stakeholder map specific for the Dutch health care system. In the interviews that followed, these stakeholders also provided more potential stakeholders that were relevant for the project, and so a specific stakeholder map appeared. Later on, this stakeholder map was used to report several business model opportunities to the management of the health IT company.

## Cocreation

Cocreation in eHealth has already been introduced in disease management, for example, to streamline health care activities among multiple health care organizations. It also plays a role in patient empowerment, as patients are actively involved in their care [12]. Introduction of eHealth technology is often top-down, that is, technology is mainly determined by management. Obviously, management has an important say in whether or not a technology should be introduced, but in our view, a bottom-up approach is needed as well. This bottom-up approach can mean, for example, that a few specialists from a hospital ward also supply input on how they see technology adding value to their work. This is *value specification* that looks further than human-centered design, as it does not only look at the usability of the technology but much wider, that is, at the intended purpose of the technology and its fit in practice.

Participation of stakeholders in development also involves a political element, in that stakeholders feel they really contribute to the technology, and therefore, they feel more involved and positive toward it than when they are excluded. Dialogue is very important in cocreation [26]. Also, scalability problems can be tackled with business modeling by planning ahead through involving future stakeholders, particularly political or influential stakeholders, early in development to avoid eHealth technology becoming too localized and too narrowly focused.

Cocreation and dialogue with stakeholders requires a willingness to be open with each other. Openness is a way of thinking that is rooted in the opportunities of open source software and Web 2.0 that advocates operating with open systems for mutual benefits and transparency [5]. The *o* *pen business model*, as described by Chesbrough, combines this idea of openness with business models and promotes that organizations can embed cocreation and collaboration in their business models for shared benefits [27]. Classic success stories of open business models are the Philips Senseo coffee machine or the budget airline Ryanair. In the eHealth context, open systems are emerging too, such as interoperable electronic health records. Business modeling also pursues openness as multiple organizations cocreate value of technology and share benefits.

Regardless of the industry, traditional boundaries between organizations are becoming fuzzier and open business models pave the way for future collaborative success.

When cocreation is a goal, it will mean that eHealth technologies will be more intricate than one single organization carrying full responsibility, and it will require cooperation of multiple health care organizations. Interorganizational dependencies can be very complex, so exploring benefits and value needs is a complex task that requires input from all involved stakeholders. To cooperate and balance these value needs, health care organizations need to extend beyond their traditional boundaries. This implies a different view of the development process of eHealth technology as well: it is not only an “apparatus” that is being created; there is a whole new underlying infrastructure for collaboration that has to be created as well (see Textbox 4 as example).

Eysenbach [8] observes that social networks, collaboration, and active participation are key elements in today’s eHealth. When the opportunities of Web 2.0 technology are used for this collaboration in eHealth, this is often called Health 2.0 or Medicine 2.0. For cocreation and collaboration, an infrastructure such as a social network of organizations is needed as well [26]. Within this infrastructure, stakeholders have to interact to cocreate value to eHealth technology. The stakeholder network that appears in the development process is also the basis for an infrastructure and will eventually become an infrastructure required for the collaboration and cocreation supporting the eHealth technology. This cocreation and collaboration is ongoing; therefore, it is imperative stakeholders all stay involved and interested in supporting and further developing the technology.

**Example Case: a Service Model for Teledermatology**

In a teledermatology project, it became apparent that the stakeholders required more than just a technology for a fitting teledermatology solution, they also required a new infrastructure for a service delivery that, for example, would replace hospital care with home care. Via stakeholder meetings, the possibilities were identified, and scenarios were made that would allow cocreation and collaboration with third parties to implement the technology in practice. This resulted in a service model that described value cocreation between the engineers of the technology company and several health service companies, which was quite different to what the management initially had in mind during the early stages of the project.

## Value Drivers in eHealth

Chesbrough emphasizes the importance of an implementation by stating that “a mediocre product with a good business model yields more value than a good product with a mediocre business model” [27]. So, business modeling is crucial for the success of an eHealth technology. Through business modeling, the entire development becomes stakeholder-focused and value-driven. Stakeholders are asked early on what value drivers they expect regarding eHealth technology. These value drivers are relevant for both the design of technology as well as the *design* of the implementation strategy that will determine effectiveness and sustainability of eHealth technology.

Business modeling is a value-driven process and, as such, it is not simply a business model but an extensive process through which early opportunities for an eHealth technology are explored, assessment is made of what is required, a case-specific business model is developed, and the said technology is accordingly implemented. As part of the roadmap, we stress that development is a continuum and thus requires ongoing research activities that include design, evaluation, and redesign. Making a choice based on facts today can be improper a week later when new facts emerge. Web technology in particular is notorious for being relentlessly progressive; thus, adaptability is crucial. Over time, stakeholders can come and go or their value needs change, and the implementation needs to be reevaluated and redesigned. In terms of business models, this is called *business model erosion* [28], and due to this erosion, eHealth technology will be less sustainable and effective. So we need more sustainable methods to ground the eHealth development process and, for this, stakeholders need to be continuously involved in the development process and have their say in an implementation.

Our current approach to business modeling is to hold various workshops with relevant stakeholders to determine problems and opportunities in health care, which role technology can play, and which stakeholders are involved and what their importance is to the developed eHealth technology. Stakeholders at the workshops determine the role that the technology needs to fulfill in practice by forming an infrastructure and also determine what makes or breaks effectiveness and sustainability. All these elements are captured with a business model that can be detailed in a business case for further operationalization and deployment of the eHealth technology.

Value creation is central to business modeling. Obviously, in for-profit contexts, this value is mostly monetary, but other kinds of value drivers can be important too. Especially in the health care context, we often see extra attention paid to nonmonetary values, as health care is a special market. Intel’s health care information technology (HIT) value model breaks down value into three levels: monetary value, quantifiable value, and benefits, the latter being, for example, social value or certain qualitative values that are considered beneficial but are hard to express in concrete figures [29]. In our business modeling approach, value drivers can be seen very broadly, that is, anything that a stakeholder considers critical to technology is a relevant value driver worthwhile to research. These values drivers form the basis for the development process and implementation.

Business modeling promotes a value-driven dialogue and promotes better understanding of what should be accomplished with eHealth technology [30]. This value-driven approach allows stakeholders in eHealth technologies to better discuss and reflect on the intended value that technology has to offer to the health care setting. Value drivers can also be initially counterproductive, as, for instance, when a certain stakeholder loses money or influence, this stakeholder will then criticize the technology. These negative value drivers then must be compensated for elsewhere. Also, by determining the overall expected value before designing begins, the assessment will be more profound whether or not eHealth technology is worth the investment. Nevertheless, *value* and *value drivers* remain complex concepts. During the value specification, many values will appear and many will also conflict; hence, dialogue is very important. It can be an extensive task to assess and to clarify to stakeholders what value eHealth technology can create, but without looking into value drivers, exact gains of eHealth investments remain unclear *a priori*, and it will be impossible to find a fitting implementation.

With business modeling, we aggregate all value needs *bottom-up* from the stakeholders, and, through dialogue, we try to cocreate a fit between all the values that will become the overall expected value of the eHealth technology. Value becomes the focal point for technical design and also for the critical success factors [31] required for implementation. In our workshops, we use custom mapping software, to elicit these values from stakeholders and to rank scores to their importance according to the stakeholders. This ranking acts as a way to quantify and prioritize values. (A common method for this is called the analytic hierarchy process [32] that, in short, alters the initial scores given to the values by taking the hierarchy of these values into consideration.) These values are input for the design of an eHealth technology and are the basis for implementation. For example, if the value *security* is given a high score by multiple stakeholders, then during implementation, all security-related choices (eg, collaboration with a good software security company) need to be given serious consideration; otherwise, certain stakeholders will not consider the technology valuable. This determination also influences the technology itself, that is, security-based features are apparently important, and thus designers and developers should thoroughly research what the security requirements are. Textbox 5 provides another example.

**Example Case: How Value Drivers Can Influence Technical Design**

During the problem analysis in the teledermatology project, it was found that there were many additional problems in the whole teledermatology process that the initial design of device did not reflect. In general, the device had to offer support regarding how health care professionals in home care can take pictures of wounds so that wounds can be better diagnosed. Consensus arose among stakeholders that it was necessary to provide standardized guidelines for using the technology. We determined what value drivers were relevant to these guidelines, as without these standardized guidelines, the device would be less useful and thus less valuable to the stakeholders. This process also resulted in technical design additions.

## Business Models

As the term *business modeling* implies, its core output is a business model. A business model plays an important part in implementation: it acts as the basis for discussion of value drivers with stakeholders and becomes the basis for further operationalization where the business model is made more concrete through a business case, and, subsequently, the actual deployment of eHealth technology can happen.

Research in business models is relatively new, and, thus far, the term *business model* is still ambiguous in science and in practice [30]. Business models are quite often confused with *business process models* that are used on an operational level to describe detailed operational processes [33]. Also, some people associate business models with detailed financial prognoses, which are actually more characteristic of a *business case*. Osterwalder [34] defines a business model as “the rationale of how an organization creates, delivers, and captures value.” By this definition, business models act on a strategic level and can be the basis for more detailed business process models and business cases [35]. In our view, one needs to decide on a business model first in order to develop a business case. The business model can be created early on in the development process. The business case can gradually take shape and the details can be developed while the technology is being designed. Obviously, during the development process, a business model can also be refined or altered depending on unforeseen changes or new insights.

Business models became prominent in the late 1990s when the methods of doing business rapidly grew more complex and interdependent [36]. During that time, Internet-based activities became important assets in value creation and opened possibilities for new moneymaking activities and sped up globalization. Organizations had to change their existing strategies and develop new strategies. Yet, in order to achieve this transformation, organizations required something to plan ahead. This is when the term *business model* became widely adopted. A business model helps to relate all strategically defined critical success factors (critical elements in the achievement of successful value creation) into a working whole [37,38]. As such, they allow managers to understand, communicate, and evaluate the strategy for value creation and to conceptualize the strategy in a concise, modeled form [37]. In this period, numerous new business models emerged, and, coinciding with the popularity of the Internet, these were, in particular, business models that explored the potential of Web 2.0 [4].

**Figure 2:**
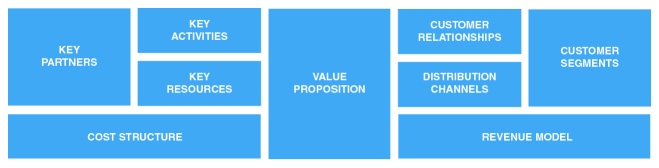
Business model canvas.

A framework that is currently popular for defining a business model is the business model canvas by Osterwalder (depicted in Figure 2) [34]. It describes the whole rationale in nine building blocks. In the middle block is the value proposition, the eHealth technology in this case. The top three blocks on the left-hand side of the diagram deal with the required organizational aspects, that is, the key activities, resources, and partners. The top three blocks on the right-hand side deal with who the customers/users are and how to interact with them. At the bottom are the financial aspects. Creating and offering value generate costs, and a revenue model is necessary to capture value back to at least cover these costs. This canvas is an empty framework or blueprint that can be filled with critical success factors and choices to describe the implementation of an eHealth technology. The framework is useful as it describes the entire value creation logic and is a guide for making sure that all nine aspects necessary for value creation are addressed. The framework also helps to classify and group the components of a business model.

However, the process behind filling this canvas determines the quality of the business model. In Osterwalder’s book, *Business Model Generation* [34], a strong focus is on *ideation* *,* that is, thinking up innovative business models on a very high level of abstraction early on for new businesses. But the canvas can also be filled with value drivers based on the value specification that we apply in our business modeling approach. The chosen, important value drivers from the value specification become critical success factors, as they will determine the success of the implementation of the eHealth technology. We place these in the canvas to get an overview as well as to check if all building blocks received adequate attention from the stakeholders and/or researchers. It is also possible that multiple business models can be formed based on the value drivers gathered from the stakeholders, as the example in Textbox 6 demonstrates.

**Example Case: Multiple Business Model Opportunities for Different Scenarios**

The aforementioned service model in the teledermatology example (Textbox 4) resulted in multiple possible business models with different service paths. These were:

· Keeping everything in-house

· Cocreation with third party organizations that would take care of the teledermatology infrastructure so that the technology company could focus on the technology

· A mix between providing a technology to third parties yet also providing additional technical services to third party organizations in return for a payment for each use

Each business model had its pros and cons, and it was up to the management to decide which of these models they found best fitting to the future of their company.

## Business Case

Having a business model alone is not enough. Once the desired business model is decided on and all stakeholders agree on the plans, the *operationalization* can be further determined by making a concrete business case based on the business model. A business case contains much more concrete information about the details of the implementation than a business model, but a business model is required to provide an idea of what the implementation should look like. In the business case, concrete descriptions of the necessary activities, resources, and costs can be written down. Usually business cases contain several financial prognoses based on estimated usage of the technology. These prognoses are based on multiple usage scenarios (low, projected, high usage) to gain a better understanding of the dynamics of the costs and potential revenues. Textbox 7 demonstrates how a business case can be made early in a project to demonstrate financial benefits of an eHealth technology. Usually, a business case is continuously updated during the development.

Also in this stage, the required infrastructure that resulted from the stakeholder network and value specification can be further arranged more formally with contracts, formal agreements, and so forth. 

Once these steps are taken and the technology is designed, it can be implemented in practice. However, the operationalization is not an endpoint; evaluation is necessary to track whether the technology and implementation still meet the intended goals and whether redesign iterations are necessary in the development.

**Example Case: Business Case for Implementing an Antibiotic Stewardship Program**

Changing antibiotic prescription can be beneficial. For example, patients can have a shorter length-of-stay or the prescriber can choose a quicker swap from intravenous to oral antibiotics. Through a calculation, we showed the hospital management that they could save up to a million euros a year on antibiotic costs alone. These financial prognoses convinced the management to start a pilot project for an antibiotic stewardship program.

## Evaluation

Development of an eHealth technology starts with a variety of assumptions defined by time or budget constraints. Not everything in a business model can be understood *ab ovo* and requires reflection and progressive insight [39]. By spending more time investigating the exact value needs—even during usage of a technology—the technology and its implementation can be continually refined. As with any technology, eHealth technologies are subject to environmental and contextual changes. Technology never stands still, and most technologies are developed using iterative design approaches [21]. Just as technologies evolve over time, business models are also not static objects [40]. Therefore, *summative and formative evaluation* cannot be performed in an inert state but should be an action or a process (see Textbox 8). Business modeling makes sure technology and implementation keep reflecting on the current and future needs of the stakeholders for sustainability. It is imperative that an eHealth technology remains an object of study even after the technology has been implemented into practice; eHealth technology is not a “fire-and-forget” technology. The evaluation of its success needs to continue for further improvement and anticipation of changes in the health care environment. As a value-driven approach can project the critical success factors, the intended goals of the eHealth technology can be measured.

**Example Case: Summative Evaluation of Web-Based Infection Control System for Methicillin-Resistant Staphylococcus Aureus (mrsa)**

In 2008, we launched a website that informs general audience and health care professionals about methicillin-resistant staphylococcus aureus (MRSA). With server logs, we analyzed how the website has been used by visitors over the years and discovered that the chosen card-sort presentation of questions and answers, codesigned in 2008 with the intended users, was indeed effective and could be maintained. Additionally, we found a few ideas for improvements such as improving the search engine optimization, as the number of visitors via Google was significantly growing over the years.

## Conclusion

Many eHealth technologies still fail in practice, and little or late attention is given to implementation. We believe preparing the implementation strategy is part of the development process and should start as early as possible in the development. In strategic management, business models are used to define the rationale behind value creation in terms of eHealth, which means the required rationale for implementing an eHealth technology in its care setting. We introduced business modeling as a vital part of our holistic approach for eHealth development in order to improve the uptake and sustainability of eHealth technologies. Business modeling, and our CeHRes Roadmap generally, have proven in multiple, different eHealth projects to be worthwhile in the development of eHealth technologies, helping us to find a better fit among humans, organizations, and technology with a value-driven and stakeholder-focused eHealth development. Business modeling fosters a ground for dialogue regarding the perceived value and purpose of an eHealth technology. An eHealth technology simply has a plethora of stakeholders and they all influence or are influenced by the eHealth technology. Implementation of eHealth technology depends on how well the value needs of stakeholders are met and how they partake in the infrastructure needed for the eHealth technology. Business modeling is a continual activity because the environmental conditions in eHealth are dynamic, so iterative development and anticipation to changes are important for sustainability and long-term success of the technology.

Health care organizations base their operations on century-old reimbursement business models [3]. Progress in medical and technological possibilities and many sociopolitical factors have altered the processes but left settled business models unchanged. Lagging legislation, financial complexity, and a status quo of roles and dependencies seem only to work in favor of perpetuating these inefficient health care processes. Evidential benefits from eHealth technologies remain unsure, as new technological possibilities often cause extra side processes rather than an efficient replacement for the processes that need to be improved. eHealth should not be an irrelevant remake of old processes. Innovative eHealth business models require that core conceptions, current roles, and processes are reevaluated and overhauled from complex organization-centered health care chains to efficient patient-centered health care networks in which multiple health care organizations collaborate to provide care.

eHealth projects need to research new business models. Both in practice as in academic context, a business model is often mentioned as a kind of panacea to improve the effectiveness and sustainability of eHealth technologies; however, the exact *why* and *how* are omitted from the arguments. Often generic business models from other industries (at the so-called taxonomy level) are mentioned as potential solutions which are *per se* unsuited, for example, taxonomies such as subscription-business models or pay-per-click-business models. These generic business models are excellent for classification, but for implementing an eHealth technology, this level-of-detail will not suffice. It is possible to inspire business models from other industries for eHealth, for example, in 2000. Parente described four e-commerce-inspired eHealth business models that were emerging at that time along with the growth of e-commerce generally [41]. E-commerce activities are probably easier to mimic from other industries than business models for health services and their complex value cocreation activities.

Not only are new business models for eHealth needed but also needed are the approaches for creating them. Admittedly, the lack of publications that discuss *how* business models can be created is not only a problem in eHealth. In general, few approaches to defining business models exist or remain cursory. Another barrier is the problem of introducing business-like thinking in health care. This continues to be a sensitive topic, as in the field of health care, the focus is the well-being of patients; thus, focusing on money is considered in a negative light because it is not patient-centered. However, with the emerging problems that health care is facing, business-like thinking could be pivotal in keeping quality health care affordable.

## Future Research

We have applied and are applying the CeHRes Roadmap in several of our eHealth projects, which are all quite varied and exist in different settings ranging in complexity and size, yet all of these projects are focused on providing some form of technology that supports disease management. A few example projects that have made or are currently making use of the roadmap and, therefore, also of business modeling are shown in Textbox 9.

**Examples of Projects Using the CeHRes Roadmap**

· Collaboration platform for cross-border infection prevention

· Setting up an antibiotic stewardship program

· Development of a teledermatology device

· Personal assistance website for diabetes care

· Prevention and quick warnings regarding the dangers of Lyme disease

All of these cases are useful for testing and improving the roadmap and are relevant to this paper. They are test cases for the current instruments for business modeling. We see that the roadmap and business modeling are applicable in all these different types of eHealth technologies, and we are working on adding instruments and evaluating current instruments. In a subsequent paper, we will give an introduction to these instruments and how they can support eHealth development. Our goal is to find robust instruments that are generic enough to be applicable for all eHealth technologies. Thus far, we have seen with our current focus groups and workshops as well as with our mapping tools that the extra effort of business modeling gives vital information not only for the implementation but also vital information with consequences for the design of the eHealth technology.

We also plan a systematic review to predetermine outcomes and effects of interventions in the antibiotic stewardship programs. After this review, we hope to assess how a literature review can be used as input for the start of the value specification by providing the outcomes and effects as general value drivers to discuss with the stakeholders.

The roadmap has been made public as a wiki (ehealthwiki.org). The goal is to provide a platform for anyone interested to collaborate on providing methods, ideas, and example cases for eHealth development as described by our roadmap. Obviously, we would also like to see contributions to the business modeling side of the roadmap.

## Abbreviations

CeHRes: Center for eHealth Research and Disease Management
HCD: human-centered design
HIT: health care information technology
IT: information technology
MRSA: methicillin-resistant staphylococcus aureus
